# An Update of Clinical, Epidemiological, and Psychosocial Features in Gamma-Sarcoglycanopathy

**DOI:** 10.3390/muscles2020012

**Published:** 2023-04-03

**Authors:** Naoufel Chabbi, Corrado Angelini, Alicia Aurora Rodriguez

**Affiliations:** 1 Abulkacem Chebbi Medical Center, Tunis 1089, Tunisia; 2 Campus Pietro D’Abano, University of Padova, 35128 Padova, Italy; 3 Neuro-e-Motion Research Team, Faculty of Health Sciences, Department of Psychology, University of Deusto, 48007 Bilbao, Spain; aliciarodriguez.b@deusto.es

**Keywords:** limb-girdle muscular dystrophy, gamma-sarcoglycanopathy, Tunisian muscular dystrophy, quality of life, socioeconomic impact

## Abstract

Limb-girdle muscular dystrophies (LGMDs) represent a group of muscle diseases due to monogenic mutations encoding muscle proteins that are defective for heterozygous and homozygous mutations prevalent in certain regions. Advances in knowledge of their pathophysiology have shed light on these rare diseases, which were, until recently, difficult to diagnose. This paper has described the process of diagnosis in autosomal recessive limb-girdle dystrophy that in Tunisia are due to the c.521del mutation in gamma-sarcoglycanopathy and to ethnically specific mutations in other countries such as Italy. The epidemiology, pathophysiology clinical features, and the main socioeconomic needs as well as research progress are discussed. We discuss an Italian case for its psychosocial impact and socioeconomic consideration and compare this case with Tunisian patients.

## 1. Introduction

The names Severe child autosomal recessive muscular dystrophy (SCARMD) and Tunisian muscular dystrophy (TMD) were used [1] to designate a common myopathy in Tunisia affecting children of both sexes, similar to Duchenne’s myopathy, and the prevalence is high in this country [1,2]. This myopathy was denominated LGMDR5, and the Tunisian type was found to have a homozygous mutation: c.521del mutation [3].

We propose to shed light on the different facets of this LGMD by coming back to the process of distinguishing it from the cohort of hereditary LGMD.

The progress of myology over the last decades, in hereditary muscle dystrophies, identified molecular defects in LGMDs, including sarcoglycanopathies, the first of which was gamma-sarcoglycanopathy [3] or LGMD2C, later renamed LGMDR5.

The near-exclusivity of its mutation (c.521del) [4] of chromosome 13p12 in Tunisia may suggest the name of TMD for this myopathy characterized by such mutation.

The benefit of this denomination and the advantages that this will bring to patients and their families are as follows: to facilitate their management at health institutions in specific medical trials and to create an emotional link to their disease using a familiar identification [5,6]. 

## 2. Evolution of the LGMD Concept

Hereditary muscular dystrophies are common where consanguinity favored their occurrence [7]. LGMDs, including SCARMDs, have been reported among these [8,9,10]. They were very common, and in the molecular era the classification was revised [5,11].

LGMDs were described by Walton and Natrass in 1954 [12] from other known myopathic entities, Duchenne’s X-linked myopathy, facioscapulohumeral myopathy, and myotonic dystrophy. These authors defined LGMDs by weakness and progressive and symmetric muscular atrophy, primarily of the girdle, recessive or dominant autosomal heredity, with CK elevation, the myogenic pattern on the electromyogram, and muscle histology consistent with dystrophy.

This broad initial definition included disparate heterogeneous muscle diseases, often with laborious diagnosis, under the same title, a catch-all group for Michel Fardeau [13]. This mainly included girdle muscular atrophy, but also distal atrophy, as well as Emery Dreifuss and Leyden Moebius, SCARMD, or Duchenne-like.

The phenotypic spectrum of LGMDs ranged from asymptomatic cases with isolated elevation of CK to dystrophies, from recessive autosomal to rare dominant autosomal, with muscle involvement only, which is the most common, to those involving the heart, or those combining both, sometimes with bone and joint involvement or, more rarely, brain involvement.

Due to the progress in knowledge of these myopathies in genetics and biochemistry, the concept of LGMD has evolved, requiring periodic revisions to the nomenclature and classification. During the 30th–31st international workshop of the European Neuromuscular Center (ENMC) in 1995 [14], it was agreed by consensus to assign the number 1 for dominant autosomal (LGMD1), and 2 for recessive autosomal (LGMD2), followed by a letter of the alphabet in the order in which the loci of their genes are discovered. 

Other LGMD genes were discovered thereafter, with their number quickly exceeding the letters of the alphabet with up to 32 LGMD2s today and 10 LGMD1s. A revision of the nomenclature was required, and in 2017 during the 229th international workshop of the ENMC [15], R replaced the number 2 for recessive and D the number 1 for dominant, followed by the number of the LGMD, from the first discovered to the most recent, and then the protein (Table 1). To be included, the LGMDs should be reported in at least two unrelated families, having imaging showing muscle atrophy groups in a degenerative fibrofatty condition, CK levels elevated to varying extents and an appearance of dystrophy in the histological study of the muscle.

The DNA study by genetic linkage research in families with many patients screened for genomes using sequence-markers, which marked chromosomes and helped to locate suspicious genes on the chromosomes [11] through their genetic distance in centimorgan compared to the markers. Then polymerase chain reaction (PCR) sequencing techniques were used to specify the nucleotide sequences of these genes and the nature of their mutations.

The discovery of the different muscle proteins and sequences of the genes that encode them, through the PCR reverse transcription technique on mRNA coming from the muscle biopsy [16], was able to give the myopathies a molecular identity and distinguish them from each other. After dystrophin [17], a large, mutated, subsarcolemmal, glycosylated protein, deficient in dystrophinopathies, alpha sarcoglycan, 50 DAG protein, first named Adhalin from the Arabic word adhala (muscle), and then the other sarcoglycans were discovered [18].

There are six sarcoglycan (SG) proteins in the striated muscle: SG-Alpha, SG-Beta, SG-Gamma, SG-Delta, SG-Epsilon, and SG-Zeta. In addition, SG-Beta and SG-Delta are expressed in the smooth muscle of the coronary vessels; their mutations more frequently lead to heart disease. Epsilon and Zeta do not cause muscular dystrophies, while the epsilon SG mutation is associated with hereditary myoclonic dystonia [19]. Their molecular weights are 35 KD for Gamma, 50 KD for Alpha, 45 KD for beta, and 35 KD for Delta. They are encoded by four genes, SG-Gamma, SG-Alpha, SG-Beta, and SG-Delta genes located on four different chromosomes, ch13q12, ch17q21.33, ch4q12, and ch5q33.2, respectively.

**Figure 1:**
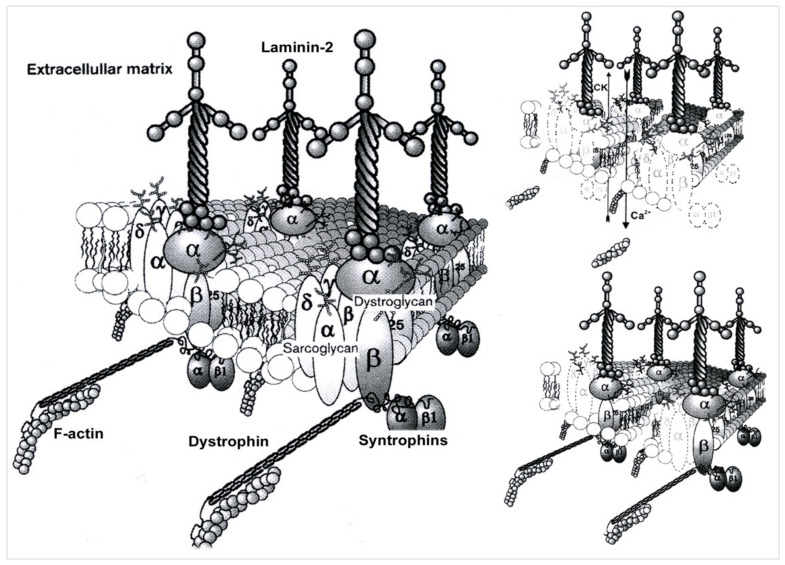
Pathomechanism of gamma-sarcoglycanopathy. Left side: In skeletal muscle, the dystrophin-associated glycoprotein complex forms a link between the actin cytoskeleton and the extracellular matrix that is critical for muscle integrity. Within this complex resides the sarcoglycan subcomplex, which consists of four transmembrane glycoproteins (alpha-, beta-, gamma-, and delta-sarcoglycan). During its assembly, beta-sarcoglycan tightly associates with delta-sarcoglycan to form a functional core that then recruits gamma- and alpha-sarcoglycan to form the sarcoglycan complex. Upper right side: mutations in γ-Sarcoglycan (35 kDa) determine gamma-sarcoglycanopathy; this condition determines sarcolemmal instability, resulting in Ca++ ion entry and CK efflux from muscle fibers. Lower right side: an important feature of sarcoglycanopathy is the close association of the four transmembrane glycoproteins, which determines the loss of the whole sarcoglycan complex from the mutation of one of the glycoproteins.

**Figure 2:**
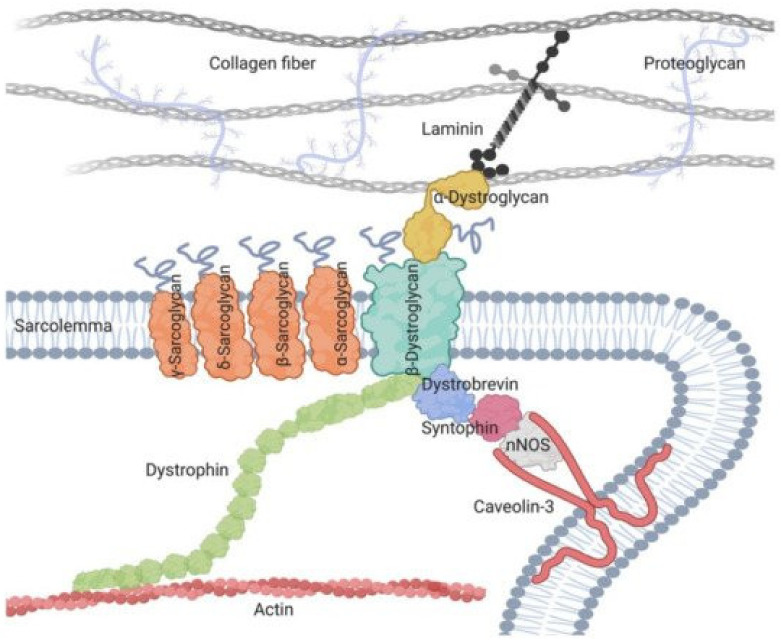
Sarcoglycan complex in sarcolemma.

The muscle sarcoglycans represent a subset of small, glycosylated, trans-sarcolemmal interlinked proteins, associated with dystrophin through another subset of glycoproteins, the dystroglycan alpha and beta. This led to the concept of the dystrophin-associated glycoprotein complex which sits across the sarcolemma, ensuring its integrity by linking sarcomeric actin to laminin alpha 2 of the extracellular matrix, thus causing a linking effect of the membrane during muscle fiber contractions [20]. The four sarcoglycans function as a tetrameric unit, with one mutation in one of their four genes, secondarily but variably affecting the expression of the three others [20]. In skeletal muscle, the dystrophin-associated glycoprotein complex forms a link between the actin cytoskeleton and the extracellular matrix that is critical for muscle integrity. Within this complex resides the sarcoglycan subcomplex, which consists of four transmembrane glycoproteins (alpha-, beta-, gamma-, and delta-sarcoglycan). During its assembly, beta-sarcoglycan tightly associates with delta-sarcoglycan to form a functional core that then recruits gamma- and alpha-sarcoglycan to form the sarcoglycan complex. The pathomechanism of gamma-sarcoglycanopathy is illustrated in Figure 1 and Figure 2. 

In Tunisia, M.Ben Hamida had reported on the frequency of a myopathy affecting girls and boys and resembling Duchenne myopathy, as SCARMD [11]. In 1992, international collaborative work with Noguchi [3] placed the locus on chromosome 13q12; this myopathy would be classified as LGMD2c. Then in 1995 [3], it was assigned the name of Gamma-sarcoglycanopathy from the name of the 35 DAG protein that encoded it, and its LGMD2C c.521del mutation was highlighted. 

This mutation affects almost all Tunisian LGMD patients, coming from a private foundational event [1], which means a foundational event in a family or a population where consanguinity is present. However, rare cases have been reported elsewhere [6]. SCARMD cases originated from the southern Mediterranean region before being linked to Gamma-sarcoglycanopathies, which were once considered adhalinopathies. This deficiency was eventually found to be secondary to a gamma-sarcoglycan deficiency. 

The DNA and mRNA sequencing transcribed from this LGMD was due to mutations of the sarcoglycan genes [21,22]. These are very numerous, of small size in most cases, and of various mechanisms, often missense, sometimes nonsense, often with sights of the reading frame. They are the cause of sarcoglycanopathies, which are homozygous with two identical mutations on the two alleles, or compound-heterozygous with two different pathogenic mutations on each of the two alleles. 

The biochemistry and bioinformatics predicted the morphology of the sarcoglycan protein, their primary amino acid sequences, tertiary arrangements, domains, locations, contact and signaling areas, and their functions, as well as the protein and clinical consequences of various nucleotide mutations. All of these advances in knowledge have led to new ways of classifying them by their defective proteins, the locations in the muscle, and the functions of these proteins [23].

## 3. Epidemiology and Psychosocial Consequences of LGMDs

The first epidemiological studies of LGMDs and sarcoglycanopathies were difficult due to the rarity of the cases, variability of the prevalence of LGMDs in different countries, disparities in health system levels, non-standardized clinical studies using different methodologies, and different patient assessments, first done by Western blot biopsies. Cohorts of patients then benefited from increasingly efficient genetic identification employing next-generation sequencing tests (NGS) [18,22,24]. 

Epidemiological analyses then became more reliable using molecular diagnosis, databases [25], national registries, international collaboration, the creation of cohorts, systematic reviews on LGMDs; meta-analyses can be inferred from these.

The most recent epidemiological studies are a contribution to the diagnostic strategies, genetic counseling, phenotypic prediction, and clinical trials on LGMDs as well as the assessment of the economic and social consequences of these diseases.

Data collection has become increasingly standardized. These are sometimes specific to different populations; they will be recorded in a national LGMD registry, that gathers the clinical and natural history, MRI, protein, and sequence in databases, all following the written consent of the patient or their relatives [26].

The data banks are used to store the vast mass of information on LGMDs from all over the world and enable it to be rapidly analyzed. This information is thus made available online to physicians, researchers, the pharmaceutical industry, and patient groups. Some data banks are for shared clinical use, such as OMIM (Online MENDELIEN inheritance in man) [27], LMDp (Leiden muscular dystrophy pages) [28], and ORPHANET (Orphanet rare diseases), while others are highly specialized.

Multiple epidemiological characteristics have thus been highlighted in LGMD by several studies: prevalence, relative frequencies of groups and subgroups, phenotype-genotype correlations, etc. 

Considering LGMDs globally, their prevalence in the general world population would be between 1/14,500 and 1/123,000 [29]. The proportion of recessive forms of LGMDs, significantly greater than the dominant forms, appears to be an item of data common to all studies [30,31]. The proportion of sarcoglycanopathies among LGMDs varies from one country to another [32]: 8.8% in Japan, 25% in the Netherlands, and 55% in Brazil. 

The prevalence of all sarcoglycanopathies worldwide would be from 1/178,000 to 1/370,000 according to Angelini [5] and represent 10 to 25% of all LGMDs. The proportions of different sarcoglycanopathies, among the sarcoglycanopathy subgroup, vary according to studies from various countries. In a series of 332 Italian and American non-Duchenne myopathy patients [16], 9% were LGMDs. Among these, sarcoglycanopathies were represented by 22% in Duchenne-like cases, 6% in late-onset cases, and 0% among congenital cases. Among 370 genetically identified LGMDs in Italy [31], 16% were LGMD1 dominant autosomal and 84% were recessive autosomal; the latter consisted of 24.7% of LGMD2A, 23.8% of LGMD2B, and 20.1% of LGMD2C, D, E, F. 

According to the French association of myopathies in 2020 [27], LGMDs are, in order of frequency, calpainopathies (LGMDR1), anoctaminopathies (LGMDR7), dysferlinopathies (LGMDR2), and then sarcoglycanopathies (LGMDR3,4,5,6). This order closely corresponds to the date of discovery of their genes. In a recent multicenter study in Brazil, among 305 families with LGMD, 30% had LGMDR1, 30% LGMDR2, and 21% LGMDR3–6. Among sarcoglycanopathies in Europe, alpha is the most common and delta is the rarest. In north Africa and in the European Roma population, gamma-sarcoglycanopathy is the most common LGMD [18,33].

In Tunisia, the predominant LGMDR is gamma-sarcoglycanopathy almost exclusively due to its c.521del mutation [4]; other LGMDs have been reported more rarely [8,9,10]. This recessive autosomal myopathy seems to be more common than Duchenne’s myopathy [5,20], but this cannot be confirmed with certainty without a molecular diagnosis. 

The molecular identification rate has been tested in LGMD cohorts. It is used to compare the efficacy of different diagnostic strategies. Thus, among 4656 cases of LGMD [24] who underwent next-generation sequencing (NGS) tests, genetic identification was possible in 27% of cases, 17% of which were LGMD2A, 17% LGMD2B and 3% sarcoglycanopathies, LGMD2C-D-E-F. Other strategies will therefore be necessary for undiagnosed cases. 

The allelic frequency of a pathogenic variant of LGMD is also an essential item of epidemiological information. It is available in genomic data banks of populations [23]. LGMD allele testing is used to detect healthy carriers and enables the statistical calculation and prediction of the LGMD prevalence in populations [23]. The frequency of the SG-Gamma c.521del pathogenic allele in a control sample of the Tunisian population [1] does not differ from that of the control sample of Caucasian Americans. The high frequency of Tunisian patients could be explained by strong consanguinity with a founder effect of the c.521del mutation demonstrated through the segregation of nearby SG-Gamma gene alleles in patients and their relatives.

## 4. Pathophysiological Data for LGMDR5

The SGCG gene, whose mutations are responsible for LGMDR5, was located in chromosome 13q12 [1] and then identified [3]. It consists of eight exons with 144 KB [34]. The mutation responsible is a homozygous mutation on exon 6 by point deletion of a c.521del [3]. This nonsense mutation creates an amino acid substitution in the protein (p.leu174argfs) and shifts the reading frame from codon 157, creating a premature stop codon on codon 193. A truncated protein is thus produced that lacks the distal part of its extracellular terminal C end.

More than 80 other mutations were discovered on this gene [21,22]: homozygous or compound-heterozygous, often small and isolated, other larger exonic mutations, or deletion of the entire gene. These mutations are available in the database (https://www.omim.org/entry/608896, accessed on 13 October 2022). Their prevalence varies depending on the population.

The researchers thus specified the topology of the genomic mutations in the sarcoglycan protein domains and correlated them with the clinical phenotype. The site and mechanism of a mutation on an SG-Gamma gene could influence the phenotype of the disease, such as its severity or even its association with heart disease. However, variability would also depend on other factors, such as environmental or genetic factors. 

It was also shown that a homozygous or compound-heterozygous pathogenic mutation on one of the four sarcoglycan genes led to the dysfunction of the sarcoglycan complex [35] and also to that of the associated dystrophin-glycoprotein complex, resulting in a final pathological process common to that of Duchenne muscular dystrophy [35]: the destruction of the cell membrane, massive calcium penetration, extracellular leakage of muscle enzymes, then degeneration of muscle fiber, necrosis, phagocytosis, etc. 

**Figure 3:**
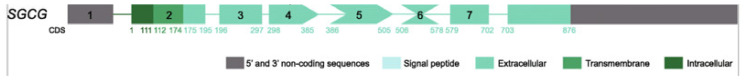
Gamma sarcoglycan molecule, showing exon and intron sequence, modified from [22].

The wild protein encoded by the SGCG gene is formed by 291 amino acids (AA), with a trans-sarcolemmal location, a large extracellular C-terminal domain that plays an important cytoarchitectural role in conjunction with the three other sarcoglycan proteins of the sarcoglycan complex and with beta-dystroglycan (Figure 3).

In the post-translational phase [36], sarcoglycans are synthesized in the endoplasmic reticulum (ER) and arranged in their tertiary structure (folding). Here, they undergo the necessary glycosylations for their sarcolemmic functions, and then leave interlinked the (ER) for the Golgi apparatus where their maturation is completed, and from where they come out linked to the beta-dystroglycan protein to join the sarcolemma by vesicular trafficking where they assume their position within the associated dystrophin-glycosyl protein complex.

The residual altered proteins are destroyed by a proteolytic quality-control system or endoplasmic reticulum-associated degradation, ERAD. The inhibition of this proteolysis makes these proteins more available to migrate to the sarcolemma and thus becomes a potential therapeutic objective [36].

The composite picture of the wild gamma SG-Gamma protein, as with other proteins, was established, and the different functions of this protein are still being tested. Gamma sarcoglycan is made up of three domains: an N terminal intracytoplasmic domain (AA 1 to 37), a small trans-sarcolemmal domain (AA 38 to 58), and a large extracytoplasmic domain (AA 59 to 291) where the C-terminal function is located. 

The missense mutations, responsible for misfolding, often of a benign phenotype, or nonsense mutations, such as LGMDR5 c.521del with more or less extensive protein amputations and of more or less severe clinical phenotypes, have been located in these three domains. Signaling and contact zones with other protein structures, providing various functions, have been found along the protein, such as contact with other sarcoglycans, with beta-dystroglycan (see Figure 1) for a cytoarchitectural and sarcolemmal protection function, with sarcospan for a cytoarchitectural and signaling function [35], and neuronal nitric oxide synthase (nNOS) which plays a role in cellular ischemia [37]. The discovery of these different contact zones and functions in the protein makes it possible to advance the knowledge of the pathophysiological mechanisms of this disease and to consider new therapeutic pathways.

## 5. Diagnostic Approach

The knowledge of the clinical spectrum of LGMDR5 enables the diagnostic approach to be well-defined. It has been noted that it can affect girls or boys, with or without consanguinity with relatives, sporadic or with familial cases, and varying severity among siblings is possible.

In most cases, the clinical spectrum of LGMDR5 is non-specific Duchenne-like. It has an onset between 3 and 10 years of age, progressive deterioration of gait, loss of ability to walk before the age of 20 years, and death before the age of 30 years. Other signs appear, such as stereotype girdle deficiency, sometimes having a tiptoe gait in the early stage of the disease, with very frequent calf hypertrophy, scapulae winging, and inconstant hypertrophy of the tongue, but without facial or bulbar involvement. Joint contractures, scoliosis, and muscle wasting are observed in the late stage. Dilated heart disease, sometimes associated with restrictive respiratory damage, is the cause of limited life expectancy. The absence of cognitive impairment is a significant and distinctive factor in Duchenne myopathy [37]. More benign, Becker-like late-onset LGMDR5 cases, with the ability to walk during adulthood, are rare.

Asymptomatic hyperCKemia, or even associated with fatigue on exertion, myalgia, or rhabdomyolysis, were genetically identified as gamma-sarcoglycanopathy, but without isolated heart disease, as with the rare delta-sarcoglycanopathy [12].

Routine examinations will complete the orientation, and molecular examinations will confirm the diagnosis.

Ck levels are significantly increased, which is a non-specific sign of the muscular dystrophy phenomenon. Their level decreases in the advanced stages of the disease. 

Electromyography, by showing a typically rich, interferential, myogenic trace, with low amplitude and polyphasic potentials, helps the clinician with the diagnosis of myopathy and to rule out neurogenic, myositis, myotonic, or neuromuscular junction disorders. 

Magnetic resonance imaging (MRI) of the muscles supports the diagnostic assumption of LGMDR5 due to the appearance of muscle abnormalities [38] and their predominant distribution in the anterior compartment of the thighs. The MRI thus helps to limit the molecular search field [39,40,41].

The muscle biopsy includes various techniques: the histological study which shows non-specific muscle dystrophy lesions and rules out metabolic or inflammatory myopathies, and the immunocytochemical study, which uses antibodies against antigens of different muscle proteins. When anti-dystrophin is positive, dystrophinopathy is ruled out and LGMD is suspected, although LGMD may include a secondary decrease in dystrophin. Anti-sarcoglycan antibodies, anti-dystroglycan, and the entire panel of available antibodies are then tested. An isolated or associated absence or decrease in the alpha, beta, gamma, or delta must be interpreted on a case-by-case basis, bearing in mind that in LGMDs a protein defect may secondarily alter the expression of others, particularly among sarcoglycanopathies [4].

Muscle protein Western blots from the sampled muscle are used to optimize protein identification of LGMD. The results of this immunoblotting technique, when available, sometimes require a discussion for interpretation.

In the end, the molecular diagnosis follows a simple pathway: after a suggestive muscle biopsy, a simple PCR sequencing highlights the almost exclusive c.521del.

The availability of NGS by a panel of neuromuscular diseases, exome sequencing, or even whole genome sequencing, will in the future optimize the definitive diagnosis but also the discovery of the molecular identity of other potential muscular dystrophies. The most frequent presentation is Duchenne-like myopathy; however, variability in clinical features, such as loss of walking ability, is rather common. The most common symptoms are proximal limb-girdle muscle weakness or wasting (common in about 80–90% of cases); other frequent features are scoliosis (40%) cardiomyopathy (30%), respiratory deficiency (50–60%) and retractions (70%), which are frequently observed especially in advanced cases.

## 6. Therapeutic Perspectives

The clinical trials relating to LGMDR5 are still in the early stages. Many ongoing investigational projects explore the major research paths common to all hereditary neuromuscular diseases. In this review, we will only suggest the main priorities of the repair of gene mutations and their consequences [42]. DNA repair proceeds by viral transfer of normal or corrected genes [43,44], especially since the sarcoglycan gene is small and easily packable in the carrier virus, unlike the dystrophin gene, or even by the transfer of normal or corrected genes.

The fine and precise repair of the mutation in the genome in myoblast cells by the gene-editing technique has produced promising results [45] at the experimental stage. 

The repair of mRNA, by the exon-skipping technique, proceeds through the antisense oligonucleotides injected directly or by viral transfer. By restoring the gene, the disturbed reading frames back to their initial position, and exon skipping may lead to the recovery of an acceptable translation of a truncated but effective protein. Despite the small size of the SGCG gene, multiexon skipping [46] permits the expression of the mini-gamma truncated protein, restoring function in myoblast culture with gamma sarcoglycan c.521del in the mice. This is interesting for future clinical trials.

The ataluren molecule, an analog of aminoglycoside antibiotics, acts on mRNA by allowing the readthrough of the stop codon, used orally in nonsense mutation Duchenne muscular dystrophy cases [1]; it could in theory be tested in the nonsense LGMDR5 mutation.

Therapies targeting protein compensation pathways are in the early stages of experimentation. Some are aimed at blocking statins, a protein inhibitor of muscle trophism [47], and some at increasing the availability of sarcoglycans for the sarcolemma [48] by inhibiting, in the endoplasmic reticulum, the degradation by a quality-control system (ERAD) of mutated proteins that are poorly arranged (misfolding) or truncated but possibly efficient.

**Figure 4:**
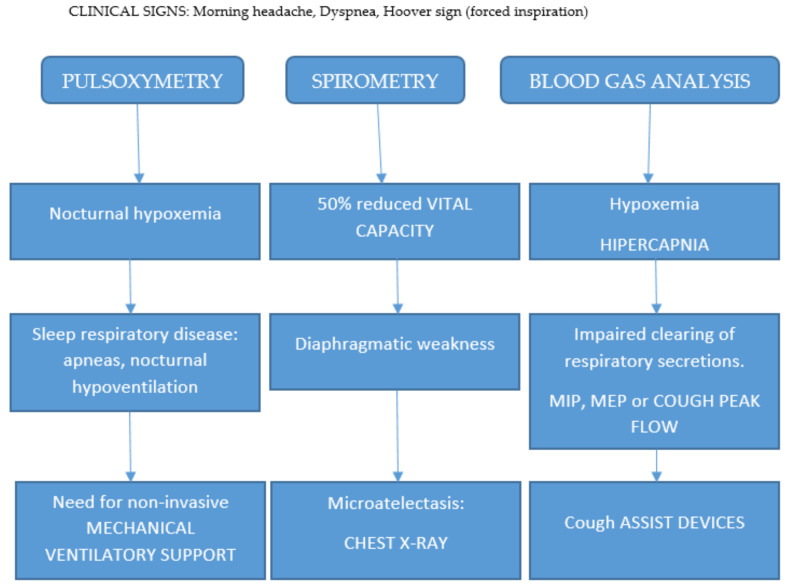
Algorithm for respiratory monitoring in sarcoglycanopathy.

There is no consensus on the use of corticosteroids in monotherapy for LGMDR5 contrary to its accepted use with Duchenne myopathy. However, it remains largely used in clinical trials in combination with genetic treatments, where it reduces their immunogenicity. Given the fact that progressive proximal limb-girdle muscle weakness, wasting, respiratory deficiency, and retractions are frequently observed in gamma-sarcoglycan-deficient cases, we propose an algorithm for the treatment of patients with a morning headache and dyspnea (Figure 4).

## 7. Socioeconomic and Quality of Life Consideration in the Case Report and Comparison between Italian and Tunisian Patients

We investigated an Italian patient to present socioeconomic considerations and the impact of gamma-sarcoglycanopathy in Italian versus Tunisian patients. This girl had normal psychomotor development and started walking at 1 year of age. The onset of the disease occurred at the age of 6–7 years when the girl walked on tiptoes. Her teacher noticed an increasing difficulty in performing physical exercises, such as hopping on one foot. CK was 24,000 U/L. At 7 years of age, EMG was myopathic and CK level was 9160 U/L. In the following year, she complained of painful cramps in her calf muscles, difficulty walking and running, and weakness in her lower limbs. She attributed her occasional falls to sudden and severe weakness in her legs. She required the use of both hands in rising from a lying or sitting position. During hospitalization, neurological examination showed waddling gait on tiptoes, difficulty on heels, positive Gowers’ maneuver, initial Achilles tendon retractions, bilateral calf hypertrophy, lumbar hyperlordosis, winging scapulae, pectus excavatum, and weakness in upper girdle muscles, neck muscles, glutei, and tibialis anterior muscles. CK values ranged from 20,000 to 6100 U/L; EMG was myopathic. EEG, EKG, and echocardiography were normal. At age 10 years, she had waddling and broad-based gait on tiptoes, calves hypertrophy, lordosis, and winging scapulae and was unable to lift her arms in the Mingazzini position. At age 11 years, she had normal spirometry and EKG and underwent bilateral Achilles tenotomy. She lost the ability to walk independently at age of 14 years, as a consequence of a fall with a consequent fracture of the rotula. At age 18 years, she could stand only when supported and had club feet, and she was unable to lift her arms. Spirometry and echocardiography were normal. At age 20 years, she had contractures of feet, knees, and hips, absent ROT, macroglossia, and marked weakness in upper and lower girdle muscles. Our analyses were done in the patient with a specific mutation (gamma); similar homozygous mutations are found in gypsy mutations and in ethnically specific mutations in Italy, which is described by Fanin et al. [49]. Therefore, we suggest using the official ENMC term of Gamma-Sarcoglycanopathy. 

Since Quality of Life (QoL) has been reduced in different research studies on the population affected by limb-girdle muscular dystrophy [50,51,52], this variable was analyzed with the INQOL instrument [49]. On the other hand, chronic diseases usually have an economic impact due to the disability produced, and the use of necessary professional services, which will depend on the health coverage of each country. The economic impact of neuromuscular diseases is well documented, so it was assessed in this case using an economic evaluation protocol developed by Rodríguez et al. [53]. The INQOL questionnaire was used to assess QoL in this patient and to analyze the specific impact. The characteristic of this disease is that muscle weakness and fatigue severely affect her QoL and severely condition her independence and activities of daily living. 

Looking specifically at this disease, the impairment of muscle weakness and fatigue have a noticeable impact on their daily life compared to other limb-girdle muscular dystrophies. These results are not surprising, considering that she lost the ability to walk at the age of 14, and that this severely affects the patient’s independence. Muscle weakness and fatigue also further impair independence, with the patient having to delegate the execution of daily activities to another person.

In terms of the economic impact of the sarcoglycanopathy, the patient uses a large part of her money on external caregivers (11,000 euros per year) and on medicines (between 100 and 500 euros per year). However, the greatest impact is due to the technical aids that the patient has to purchase as a consequence of the disease, which are listed below: anti-decubitus pillows, lifts, electric chair, adapted bed, respirator, manual wheelchair, and home automation. In general terms, and in comparison with other research carried out on the economic impact of Duchenne Muscular Dystrophy, this impact is lower in this patient. Tunisian patients do not request such an amount of money, but their care is still important, and this impacts families.

## 8. Conclusions

The pharmaceutical industry has shown great interest in LGMDs by leveraging the global network of multidisciplinary researchers to move physiopathology hypotheses into efficient therapies. These focus on the standardization of clinical tests and research on new biomarkers [54] that follow objectively the progression of the disease in natural history studies or in enrolled patients during the clinical trials.

In conclusion, it is possible to suggest that LGMDR5 c.521del is distinguished in these LGMDs as a TMD special group. Patients in this group could be included in a national registry for muscular dystrophies to prepare them for specific clinical trials with the advantages conferred by this effect and by a large number of these patients in Tunisia with this mutation. Our case shows the presence of a different homozygous mutation in the Italian population. Therefore, the name gamma-sarcoglycanopathy should be used in general. 

Awaiting potential therapy, patients should improve their QoL and life expectancy by regular follow-up during natural history studies. They will benefit from symptomatic therapies common to other LGMDs: prevention and repair of joint deformities and stiffness, detection and follow-up of heart disease, detection of impaired respiratory function and external respiratory support if necessary, and genetic counseling for the families. The patients must be made aware by genetic counseling of the risk associated with consanguineous marriage.

## Figures and Tables

**Table 1:** Sarcoglycan genes and classification of sarcoglycanopathies.

New Name	Old Name	Locus	Gene	Protein
LGMDR3	LGMD2D	17q21	SGCA	α-sarcoglycan
LGMDR4	LGMD2E	4q12	SGCB	β-sarcoglycan
LGMDR5	LGMD2C	13q12	SGCG	λ-sarcoglycan
LGMDR6	LGMD2F	5q33	SGCD	δ-sarcoglycan

## Data Availability

The datasets generated and/or analyzed during the current study are not publicly available, because they belong to the University of Padova, but are available from the corresponding author (Corrado Angelini) on reasonable request.
